# Obesity and overweight prevalences in rural and urban populations in East Spain and its association with undiagnosed hypertension and Diabetes Mellitus: a cross-sectional population-based survey

**DOI:** 10.1186/1756-0500-2-151

**Published:** 2009-07-27

**Authors:** Antonio Hernández-Mijares, Eva Solá-Izquierdo, Francisco Ballester-Mechó, María Teresa Marí-Herrero, Juan Vicente Gilabert-Molés, Natalia Gimeno-Clemente, María Morales-Suárez-Varela

**Affiliations:** 1 Service of Endocrinology, University Hospital Dr. Peset, Valencia, Spain; 2 Department of Medicine, University of Valencia, Valencia, Spain; 3 Research group CIBER CB06/02/0045, CIBER actions – Epidemiology and Public Health. Health Institute Carlos III, Madrid, Spain; 4 Official College of Pharmacists of Castellón, Spain; 5 Diabetes Unit of Hospital de la Plana, Castellón, Spain; 6 Unit of Public Health. Department of Preventive Medicine, University of Valencia, Spain; 7 Foundation for Investigation. University Hospital Dr. Peset, Valencia, Spain

## Abstract

**Background:**

An increase in the number of overweight and obese subjects in the general population has been observed.

The aim of this study was to determine the prevalence of overweight and obese subjects in the general population and its association with undiagnosed pathologies, such as diabetes mellitus [DM] and hypertension [HT], by taking age, gender and place of residence [rural or urban] into account.

**Findings:**

A cross-sectional population-based survey was conducted in Castellón, East Spain in 2005–2006. The sample included 2,062 participants aged 18–94 years. Weight, height, blood pressure and glycaemia values were recorded, and information about gender, age and place of residence was obtained. Overweight, obesity, and undiagnosed HT and DM prevalences were calculated. Multiple regression analyses were done to assess the association of overweight/obesity with undiagnosed HT and DM by adjusting for age, gender and place of residence.

The overall overweight, obesity, and undiagnosed HT and DM prevalences were 39.9% [95% CI:37.3–42.0], 25.9% [95% CI:24.0–27.9], 9.0% [95% CI:7.8–10.4] and 12.6% [95% CI:11.2–14.1], respectively. We identified various independent risk factors; those relating to overweight were increasing age, male gender and rural residential area, while that relating to obesity was increasing age. Compared to normal weight adults, the Relative Prevalence Ratio (RPR) for subjects who were overweight and had HT was 2.00 [95% CI:1.21–3.32]; that for obesity and HT was 1.91 [95% CI:1.48–2.46], and it was 1.50 [95% CI:1.25–1.81] for obesity and DM.

**Conclusion:**

Overweight and obesity prevalences, and their association with undiagnosed DM and HT, are high in our study population.

## Background

In the last two decades, the prevalence of overweight and obesity has increased worldwide [1], mainly in the adult population [2], and in both developed and developing countries [3].

In Europe, obesity prevalence rates have increased by 30% since the mid 80's [4], and are presently between 4.0–28.3% in men, and 6.2–36.5% in women. Diverse findings have been noted among various European countries: Eastern and Mediterranean countries have the highest prevalence, and Western and Northern countries have the lowest [4].

It is suspected that Spain has the highest prevalences in the European Union for both genders [4], and a geographical prevalence distribution also exists within Spain, this being higher in Southeast and Northern Spain [5,6]. A growing trend, which is in line with the rest of the world, is also noted and will imply increased associated mortality and, indeed, currently claims some 28,000 deaths a year [6,7].

This increased prevalence of obesity has also been associated with the increased risk of numerous comorbidities like hypertension [HT] [8-10] and diabetes mellitus [DM] [10,11]. A linear relationship between adiposity and HT has been noted [12]. Diabetes mellitus is a world public health priority, not only because its prevalence has sharply increased in the last decade [10], but also because of the high morbidity and mortality that it causes [13,14].

This study aims to determine the overweight and obesity prevalences in the general adult population and their association with undiagnosed pathologies [DM and HT] by taking age, gender and area of residence [rural or urban] into account.

## Methods

### Study design

A cross-sectional study was done in a general population of Castellón, Eastern Spain in 2005–2006.

### The study population

The study population included subjects aged 18 years or older and who were undiagnosed with DM and HT.

In the study area [Castellón], there are 3 Health Districts with a population of 431,093 subjects aged 18 years or older [15].

Of this population we selected 8,400 subjects by a random, stratified, multi-stage sampling technique in which the first-stage units were Primary Health Care Centres. Those with diagnosed HT or/and DM were excluded [3,503, 41.7%].

The other 4,898 subjects [undiagnosed of HT and DM] were located by telephone and invited to participate in the study. This sample size allows us an accuracy of 99.0% for an expected obesity prevalence of 15.5% [3]. The subjects who accepted invitations to participate were seen in a centre of biological analysis.

The final sample included 2,062 adults [response rate, 42.1%], 643 men [31.18%] and 1419 women [68.82%]. This sample size allows us to an accuracy of 98.5% for an expected obesity prevalence of 15.5% [3].

Participants' ages ranged from 18 to 94 years. The mean age of men and women was 58.00 ± 14.73 and 54.83 ± 14.52 respectively. Written consent to participate in the study was obtained from all the participants.

### Measurements

Specially trained professionals [clinical specialist pharmacists] conducted an anthropometric study with the participants. This study recorded weight [kg], height, and calculated body mass index [BMI] [kg/m^2^]. Those individuals whose BMI was between 25.0 and 29.9 kg/m^2 ^were considered overweight, while those with a BMI equal or over 30 kg/m^2 ^were classified as obese [7].

The participants' blood pressure was consecutively taken three times and the arithmetic mean was calculated. Diagnosing HT was conducted by following the World Health Organization's [WHO] criteria [HT: subjects with systolic pressure > 140 mmHg, and diastolic pressure > 90 mmHg] [8].

Capillary glycaemia [CG] was also determined in all the participants. Only in those participants whose CG value exceeded 106 mg/dl [if he/she had not eaten breakfast] or 140 mg/dl [if he/she had eaten breakfast], was venous glycaemia [VG] also dertmined by taking blood samples [before they ate breakfast in all the cases]. Diagnosing DM was conducted based on VG by following the American Diabetes Association criteria [DM: subjects with a VG value before eating breakfast of > 126 g/dl] [16].

Information about the participants' age, gender, and place of residence was obtained through interviews. We distinguished between places of residence as follows: rural areas with < 10,000 inhabitants; urban: areas with ≥ 10,000 inhabitants.

### Statistical analysis

Overweight, obesity and undiagnosed HT and DM [who were not on HT/DM treatment and whose blood pressure/VG exceeded the limits stated] prevalences were calculated with their corresponding confidence intervals [CI 95%]. Comparisons were done with the Chi-square test [p ≤ 0.05].

Given the sample's lack of representativeness, prevalences were estimated in the adult population of the province of Castellón by the direct standardisation procedure for samples in terms of age and gender [17] by taking the 2008 population census as a reference [15].

In order to determine the relationship of being overweight/obese with age, gender, place of residence and having undiagnosed DM/HT, a multiple regression logistic analysis was done by calculating the relative prevalence ratios [RPR], which was adjusted for age, gender and place of residence, as well as their corresponding CI 95%. Statistical analyses were done with the SPSS v.15 software.

## Results

### Overweight and obesity prevalences

The total overweight and obesity prevalences obtained were 39.9% [95% CI:37.7–42.0] and 25.9% [95% CI:24.0–27.9], respectively. By extrapolating to the adult population of Castellón, which had been adjusted for age and gender, the estimated overweight and obesity prevalences were 38.0% [95% CI:37.8–38.2] and 16.7% [95% CI:16.6–16.9] [Table 1].

**Table 1:** Age and gender stratified overweight and obesity prevalences among 2062 residents of Castellón.

	Men		Women		Total	
	Overweight	Obesity	Overweight	Obesity	Overweight	Obesity

Age Group	No % (95%CI)	No % (95%CI)	No % (95%CI)	No % (95%CI)	No % (95%CI)	No % (95%CI)

						

≤ 34	15 39.5 (25.0–55.5)	3 7.9 (2.0–20.0)	19 22.0 (14.3–31.8)	4 4.6 (1.5–10.8)	34 27.4 (20.1–35.8)	7 5.7 (2.5–10.8)

35–44	45 51.2 (40.7–61.5)	16 18.2 (11.1–27.3)	73 23.8 (19.3–28.8)	42 13.7 (10.2–17.9)	118 29.9 (25.5–34.5)	58 14.7 (11.4–18.4)

45–54	82 63.0 (54.5–71.0)	22 16.9 (11.2–24.1)	122 36.5 (3.5–41.8)	78 23.3 (19.0–28.1)	205 44.2 (39.7–48.7)	100 21.5 (18.0–25.5)

55–64	63 46.6 (38.4–55.1)	43 31.8 (24.4–40.1)	112 40.6 (34.9–46.5)	100 36.2 (30.7–42.0)	175 42.6 (37.9–47.4)	143 34.8 (30.3–39.5)

≥ 65	119 47.2 (41.1–53.4)	69 27.4 (22.1–33.1)	170 40.9 (36.2–45.6)	157 37.7 (33.2–42.5)	290 43.4 (39.7–47.2)	226 33.9 (30.3–37.5)

						

Total	324 50.4 (46.5–54.2)	153 23.8 (20.6–27.3)	496 34.9 (32.5–37.5)	381 26.8 (24.6–29.2)	822 39.9 (37.7–42.0)	534 25.9 (24.0–27.9)

Age and gender-adjusted† % (95%CI)	46.8 (46.6–47.1)	16.0 (15.8–16.2)	29.3 (29.1–29.6)	17.4 (17.3–17.6)	38.0 (37.8–38.2)	16.7 (16.6–16.9)

Overweight: Subjects with BMI = 25–29.9 Kg/m^2^

Obesity: Subjects with BMI ≥ 30 Kg/m^2^

No = Number of overweight or obese adults

† Adjusted for the age and gender distribution of the population of Castellón

### Undiagnosed HT Prevalence

Of all the participants, 186 had undiagnosed HT with a prevalence of 9.0% [95% CI:7.8–10.4]. By extrapolating to the adult population of Castellón, the estimated HT prevalence was 5.5% [95% CI:5.4–5.6] [Table 2].

**Table 2:** Age and gender stratified undiagnosed hypertension and diabetes mellitus prevalences among 2062 adult residents of Castellón.

	Hypertension			Diabetes		
	Men	Women	Total	Men	Women	Total

Age Group	No % (95%CI)	No % (95%CI)	No % (95%CI)	No % (95%CI)	No % (95%CI)	No % (95%CI)

						

≤ 34	0 -	1 1.2 (0.1–5.6)	1 0.8 (0.1–3.9)	0 -	2 2.3 (0.4–7.5)	2 1.6 (0.3–5.2)

35–44	7 7.9 (3.5–15.1)	9 2.9 (1.4–5.3)	16 4.0 (2.4–6.4)	3 3.4 (0.9–9.0)	10 3.2 (1.7–5.7)	13 3.3 (1.8–5.4)

45–54	14 10.7 (6.3–17.0)	26 7.8 (5.3–11.0)	40 8.6 (6.3–11.4)	14 10.8 (6.3–17.0)	27 8.0 (5.5–11.4)	41 8.8 (6.5–11.7)

55–64	21 15.5 (10.2–22.4)	33 11.9 (8.5–16.2)	54 13.1 (10.1–16.7)	29 21.5 (15.2–29.0)	35 12.7 (9.1–17.0)	64 15.6 (12.3–19.3)

≥ 65	30 11.9 (8.3–16.3)	45 10.8 (8.1–14.1)	75 11.2 (9.0–17.8)	60 23.8 (18.9–29.4)	80 19.2 (15.7–23.2)	140 20.9 (18.0–24.2)

						

Total	72 11.2 (8.9–13.9)	114 8.0 (6.7–9.6)	186 9.0 (7.8–10.4)	106 16.5 (13.7–19.6)	154 10.8 (9.3–12.6)	260 12.6 (11.2–14.1)

Age and gender-adjusted† % (95%CI)	6.0 (5.8–6.1)	5.1 (4.9–5.2)	5.5 (5.4–5.6)	7.3 (7.2–7.5)	7.2 (7.1–7.3)	7.3 (7.2–7.4)

Hypertension: Subjects with systolic pressure > 140 mmHg and diastolic pressure > 90 mmHg

Diabetes: Subjects with venous glycaemia before breakfast > 126 g/dl

No = Number of adults with hypertension or diabetes

† Adjusted for the age and gender distribution of the population of Castellón

### Undiagnosed DM Prevalence

Of all the participants, 260 had DM with a prevalence of 12.6% [95% CI:11.2–14.1]. By extrapolating to the adult population of Castellón, the estimated DM prevalence was 7.3% [95% CI:7.2–7.4] [Table 2].

### Gender, age and place of residence

As Table 1 shows, we saw a trend of the overweight prevalence increasing with age, which went from 27.4% in the 34 year-old and younger age group to 44.2% in the 45–54 year-old age group [p-value < 0.001].

Regarding the obesity prevalence, we observed that it increased with age, and went from 5.7% in the 34 year-old and younger age group to 34.8% in the 55–64 year-old age group [p-value < 0.001].

Table 3 shows an RPR of overweight in subjects in the 65 and older age group of 4.42 [95% CI:2.78–7.01] compared with those subjects of younger age group [34 years and below] after adjusting for gender and place of residence. For obesity, we found an RPR of 8.75 [95% CI:4.00–19.14] in subjects of 65 year and older compared to the younger subjects.

**Table 3:** Overweight and obesity prevalences and adjusted effects of potential risk factors in adults.

	Overweight				Obesity			
Risk factor	Prevalence (%)	RPR	95%CI	P-value	Prevalence (%)	RPR	95%CI	P-value

Age								

≤ 34	27.4	1(ref.)	-		5.7	1(ref.)	-	
			
35–44	29.9	1.40	0.87–2.26		14.7	2.89	1.28–6.54	
			
45–54	44.2	4.51	2.76–7.39	<0.001	21.5	4.55	2.05–10.10	<0.001
			
55–64	42.6	3.29	2.06–5.24		34.8	8.89	4.02–19.65	
			
≥ 65	43.4	4.42	2.78–7.01		33.9	8.75	4.00–19.14	

Gender								

Women†	34.9	1(ref.)	-		26.8	1(ref.)	-	
			
Men	50.4	2.06	1.62–2.63	<0.001	23.8	1.10	0.82–1.47	NS

Area								

Urban†	38.3	1(ref.)	-		26.2	1(ref.)	-	
			
Rural	46.6	1.57	1.19–2.07	<0.001	27.8	1.31	0.96–1.80	NS

Overweight: Subjects with BMI = 25.0–29.9 Kg/m^2^

Obesity: Subjects with BMI ≥ 30.0 Kg/m^2^

RPR: Relative Prevalence Ratio adjusted for age, gender and place of residence

† Referent group

P-value based on Pearson's chi-square test

NS = Non-Significant

The overall overweight and obesity prevalences for men were 50.4% [95% CI:46.5–54.2] and 23.8% [95% CI:20.6–27.3], and 34.9% [95% CI:32.5–37.5] and 26.8% [95% CI:24.6–29.2] for women. By extrapolating to the adult population of Castellón, the estimated overweight and obesity prevalences for men were 46.8% [95% CI:46.6–47.1] and 16.0% [95% CI:15.8–16.2], and 29.3% [95% CI:29.1–29.6] and 17.4% [95% CI:17.3–17.6] for women. Men of all ages tended to have higher prevalence of overweight than women. The prevalence of obesity tended to be higher in women after age 45 years and the difference was significantly higher in the oldest age group [Table 1].

After adjusting for gender and age, we observed a high overweight prevalence among the subjects living in a rural area [p-value < 0.001] with an RPR of 1.57 [95% CI:1.19–2.07] compared with those living in an urban area. For obesity, the prevalence was somewhat higher for subjects living in a rural area than for those living in an urban area, although no statistically significant differences were found [RPR of 1.31, 95% CI:0.96–1.80] [Table 3].

Table 2 shows that the undiagnosed HT prevalence increased with age from 0.8% in the 34 year-old and younger age group and to 13.1% by age 55–64 years. The undiagnosed DM prevalence increased with age from 1.6% in the 34 year-old and younger age group to 20.9% in the 65 year-old and older age group.

There was a tendency in all age groups for more undiagnosed HT and DM in men compared to women.

### The relationship between overweight/obesity and undiagnosed HT/DM

As shown in Table 4, the HT prevalence among overweight and obese participants was 10.6% and 16.9%, respectively. Compared to normal-weight adults, overweight subjects with HT had an RPR of 2.00 [95% CI:1.21–3.32], while those with obesity and HT had an RPR of 1.91 [95% CI:1.48–2.46]; both had been adjusted for age, gender and place of residence. Finally, the DM prevalence among overweight and obese participants was 9.8% and 22.4%, respectively. Compared to normal-weight adults, those who had a combination of obesity and DM showed an RPR of 1.50 [95% CI:1.25–1.81].

**Table 4:** Undiagnosed hypertension and diabetes mellitus prevalences and adjusted effects of overweight and obesity on adults.

	Hypertension				Diabetes			
Risk factor	Prevalence (%)	RPR	95%CI	P-value	Prevalence (%)	RPR	95%CI	P-value

Normal-weight†	4.2	-		-	7.8	-		-

Overweight	10.6	2.00	1.21–3.32	0.007	9.8	0.90	0.61–1.33	NS

Obese	16.9	1.91	1.48–2.46	<0.001	22.4	1.50	1.25–1.81	<0.001

Hypertension: Subjects with systolic pressure > 140 mmHg and diastolic pressure > 90 mmHg

Diabetes: Subjects with venous gycaemia before breakfast > 126 g/dl

Normal-weight: Subjects with BMI < 25 Kg/m^2^

Overweight: Subjects with BMI = 25–29.9 Kg/m^2^

Obese: Subjects with BMI ≥ 30 Kg/m^2^

RPR: Relative Prevalence Ratio adjusted for age, gender and place of residence

† Referent group

P-value based on Pearson's chi-square test

NS = Non-Significant

## Discussion

We observed a high prevalence of overweight and obesity in a Mediterranean region, and this result coincides with other studies [4]. The obesity prevalence found in both men [23.8%] and women [26.8%] is among the highest of Europe [4.0–28.3% in men and 6.2–36.5% in women] [4]. The total prevalence even exceeds that of other non-European countries like the United States [20.3%] [6]. Studies done on adults in other Mediterranean areas in Spain detected similar obesity prevalences [18]. These prevalences are higher than those described for the whole of Spain [15.5%] [3]. Therefore our results demonstrate that a geographically distributed obesity prevalence exists in Spain, which is higher in the Southeast [5,6]. Our study also determines the association of these overweight and obesity prevalences with undiagnosed DM and HT, which is new and different from these earlier published Spanish data.

Both overweight and obesity appears to increase with age. This may be due to people of this age living a more sedentary lifestyle. Besides, a series of physiological alterations take place with age which increase body weight [5].

The overweight prevalence generally tended to be higher in men than in women. However, the obesity prevalence tended to be higher in women than in men. This indicates that obesity prevention measures must address men and women equally.

Like other Spanish studies [19], higher overweight and obesity prevalences were noted for the rural population, although only the overweight prevalence was statistically significant. In the past, the overweight prevalence was traditionally higher in urban areas. However, the opposite trend applies nowadays [19]. This could be because subjects living in rural areas have poorer access to health care and they practice medical selfcare less.

These high overweight and obesity prevalences could be responsible for a possible epidemic of associated adverse effects [1,3]. Subjects with a higher BMI are at greater risk of suffering cardiovascular diseases [20-22]. An association between severe obesity and a dysfunction of the left ventricle was first observed in the mid 20's [20]. Later, clinical and necroscopic studies confirmed that cardiomyopathies were associated with obesity [22,23]. Echo-cardiography studies, catheterisation and necroscopic examination have revealed a relationship between obesity and certain alterations to both the heart structure and the systolic function in moderate obesity [23]. We detected that the undiagnosed HT prevalence, was statistically and significantly higher in overweight than in normal-weight subjects, and was even higher in obese subjects. Therefore, anyone who is somewhat over their ideal weight could benefit from a moderate weight loss in order to be at less risk of HT [24].

We also noted that the undiagnosed DM prevalence was statistically and significantly higher in obese subjects than in normal-weight subjects. BMI is an indicator of obesity and would be a possible indicator of the risk of DM [25,26].

We also observed that the prevalence of this pathology increased significantly with age, as other authors discovered [27]. Therefore, the detection of an abnormal glucose metabolism in elderly obese subjects would help control the disease. Taking healthy lifestyle habit measures has proved more effective than pharmacological treatments to prevent the onset of DM [28]. Early prevention programmes that centre on changing lifestyle habits are very important.

This study is not without its limitations. The main limitation is the sample's lack of representativeness produced by the fact that the response rate was much more elevated in women than in men. Also, for those cases in which hyperglycaemia was absent, a diagnosis of DM was not confirmed with a second test. The Oral Glucose Tolerance Test was not done; therefore the prevalence of undiagnosed DM could be underestimated.

## Conclusion

This study provides strong evidence for overweight and obesity prevalences in the general population. Besides, it demonstrates the important role that obesity plays as a main modifiable risk factor in the development of comorbidities like HT and DM and its use in early diagnoses.

## List of abbreviations

BMI: Body Mass Index; CG: Capillary glycaemia; CI: Confidence Intervals; CIBER: Networked Biomedical Research Centre; DM: Diabetes Mellitus; HT: Hypertension; kg: Kilograms; RPR: Relative Prevalence Ratios; WHO: World Health Organization.

## Competing interests

The authors declare that they have no competing interests.

## Authors' contributions

AHM had the original idea for the work and organized the study. ESI contributed to the study design. FBM provided expert advice on study organization and contributed to the writing of the manuscript. MTMH and JVGM contributed by interpreting the results and helped to write the manuscript. NGC and MMSV did the data analysis and contributed to the writing of the paper. All the authors read and approved the final manuscript.

## References

[B1] Ginter E, Simko V (2008). Adult obesity at the beginning of the 21th century: epidemiology, pathophysiology and health risk. Bratisl Lek Listy.

[B2] Avenell A, Broom J, Brown TJ, Poobalan A, Aucott L, Stearns SC, Smith WC, Jung RT, Campbell MK, Grant AM (2004). Systematic review of the long-term effects and economic consequences of treatments for obesity and implications for health improvement. Health Technol Assess.

[B3] Aranceta J, Pérez-Rodrigo C, Serra-Majem L, Bellido D, de la Torre ML, Formiguera X, Moreno B (2007). Prevention of overweight and obesity: a Spanish approach. Public Health Nutr.

[B4] Berghöfer A, Pischon T, Reinhold T, Apovian CM, Sharma AM, Willich SN (2008). Obesity prevalence from a European perspective: a systematic review. BMC Public Health.

[B5] Martínez-Ros MT, Tormo MJ, Navarro C, Chirlaque MD, Pérez-Flores D (2001). Extremely high prevalence of overweight and obesity in Murcia, a Mediterranean region in south-east Spain. Int J Obes Relat Metab Disord.

[B6] Gabriel R, Alonso M, Segura A, Tormo MJ, Artigao LM, Banegas JR, Brotons C, Elosua R, Fernández-Cruz A, Muñiz J, Reviriego B, Rigo F, ERICE Cooperative Group (2008). Prevalence, geographic distribution and geographic variability of major cardiovascular risk factors in Spain. Pooled analysis of data from population-based epidemiological studies: the ERICE Study. Rev Esp Cardiol.

[B7] Pischon T, Boening H, Hoffmann K, Bergmann M, Schulze MB, Overvad K (2008). General and abdominal adiposity and risk of death in Europe. N Engl J Med.

[B8] World Health Organization (1997). Obesity: preventing and managing the golobal epdemic.

[B9] Allman-Farinelli MA, Chey T, Bauman AE, Gill T, James WP (2007). Age, period and birth cohort effects on prevalence of overweight and obesity in Australian adults from 1990 to 2000. Eur J Clin Nutr.

[B10] Schröder H (2007). Protective mechanisms of the Mediterranean diet in obesity and type 2 diabetes. J Nutr Biochem.

[B11] Gikas A, Sotiropoulos A, Panagiotakos D, Peppas T, Skliros E, Pappas S (2004). Prevalence, and associated risk factors, of self-reported diabetes mellitus in a sample of adult urban population in Greece: MEDICAL Exit Poll Research in Salamis [MEDICAL EXPRESS 2002]. BMC Public Health.

[B12] Davy KP, Hall JE (2004). Obesity and hypertension: two epidemics or one?. Am J Physiol Regul Integr Comp Physiol.

[B13] Wee CC, Phillips RS, Legedza AT, Davis RB, Soukup JR, Colditz GA, Hamel MB (2005). Health care expenditures associated with overweight and obesity among US adults: importance of age and race. Am J Public Health.

[B14] Madden SG, Loeb SJ, Smith CA (2008). An integrative literature review of lifestyle interventions for the prevention of type II diabetes mellitus. J Clin Nurs.

[B15] Census. Instituto Nacional de Estadística [INE]. http://www.ine.es/.

[B16] American Diabetes Association [ADA]. http://www.diabetes.org/about-diabetes.jsp.

[B17] Jenicek M (1997). Epidemiology, evidenced-based medicine, and evidence-based public health. J Epidemiol.

[B18] Vioque J, Vicente MC (1994). The prevalence of obesity in Orihuela, Alicante, Spain. A comparison with the data from the National Health Care Survey in Spain. Med Clin.

[B19] Cea-Calvo L, Moreno B, Monereo S, Gil-Guillén V, Lozano JV, Martí-Canales JC, Llisterri JL, Aznar J, González-Esteban J, Redón J, PREV-ICTUS Study (2008). Prevalence and related factors of overweight and obesity in Spanish population aged 60 years-old or older. The PREV-ICTUS study. Med Clin.

[B20] Silventoinen K, Magnusson PK, Neovius M, Sundström J, Batty GD, Tynelius P, Rasmussen F (2008). Does Obesity Modify the Effect of Blood Pressure on the Risk of Cardiovascular Disease?. A Population-Based Cohort Study of More Than One Million Swedish Men. Circulation.

[B21] Gudelj-Radi J, Davidovi D, Avramovi D, Backovi D, Jorga J (2007). Factors mediating the depression in the adult obese outpatients. Srp Arh Celok Lek.

[B22] Shoelson SE, Herrero L, Naaz A (2007). Obesity, inflammation, and insulin resistance. Gastroenterology.

[B23] Pascual M, Pascual DA, Soria F, Vicente T, Hernández AM, Tébar FJ, Valdés M (2003). Effects of isolated obesity on systolic and diastolic left ventricular function. Heart.

[B24] De Luis DA, Aller R, Izaola O, Gonzalez Sagrado M, Conde R, Perez Castrillon JL (2008). Effects of lifestyle modification on adipocytokine levels in obese patients. Eur Rev Med Pharmacol Sci.

[B25] Meisinger C, Döring A, Thorand B, Heier M, Löwel H (2006). Body fat distribution and risk of type 2 diabetes in the general population: are there differences between men and women? The MONICA/KORA Augsburg cohort study. Am J Clin Nutr.

[B26] Vazquez G, Duval S, Jacobs DR, Silventoinen K (2007). Comparison of body mass index, waist circumference, and waist/hip ratio in predicting incident diabetes: a meta-analysis. Epidemiol Rev.

[B27] Crandall J, Schade D, Ma Y, Fujimoto WY, Barrett-Connor E, Fowler S, Dagogo-Jack S, Andres R (2006). The influence of age on the effects of lifestyle modification and metformin in prevention of diabetes. J Gerontol A Biol Sci Med Sci.

[B28] Waugh N, Scotland G, McNamee P, Gillett M, Brennan A, Goyder E, Williams R, John A (2007). Screening for type 2 diabetes: literature review and economic modelling. Health Technol Assess.

